# Adherence to Mediterranean Diet Pattern among Spanish Adults Attending a Medical Centre: Nondiabetic Subjects and Type 1 and 2 Diabetic Patients

**DOI:** 10.1155/2017/5957821

**Published:** 2017-12-03

**Authors:** Concepción Vidal-Peracho, José Miguel Tricás-Moreno, Ana Carmen Lucha-López, Maria Orosia Lucha-López, Ana Cristina Camuñas-Pescador, Alberto Caverni-Muñoz, Pablo Fanlo-Mazas

**Affiliations:** ^1^Department of Endocrinology and Nutrition, Hospital Royo Villanova SALUD, Barrio San Gregorio, s/n, 50015 Zaragoza, Spain; ^2^Physiotherapy Research Unit, University of Zaragoza, C/Domingo Miral, s/n, 50009 Zaragoza, Spain; ^3^Faculty of Health Sciences, Physiotherapy Research Unit, University of Zaragoza, C/Domingo Miral, s/n, 50009 Zaragoza, Spain; ^4^ALCER EBRO (Aragonese Association of Patients with Chronic Renal Insufficiency), Santa Teresa de Jesús, No. 29-35 Bajos, 50006 Zaragoza, Spain

## Abstract

**Objective:**

To identify adherence to Mediterranean diet among two groups of Spanish adults: diabetic patients and nondiabetic subjects.

**Methods:**

Adherence to Mediterranean diet was measured by a 14-item screener (scale: 0–14; ≤5: low, 6–9: moderate, and ≥10: high) in 351 volunteers.

**Results:**

Mean age was 50.97 ± 12.58 in nondiabetics (*n* = 154) and 59.50 ± 13.34 in diabetics (*n* = 197). The whole sample scored 8.77 ± 1.82. Score was 9.19 ± 1.84 in nondiabetic females (*n* = 58) and 8.15 ± 1.79 in diabetic females (*n* = 85) (*p* = 0.003), due to lower consumption of olive oil (*p* = 0.005) and nuts (*p* = 0.000). Type 2 diabetic males (*n* = 79; 8.76 ± 1.88) consumed less olive oil than healthy males (*n* = 28; 9.36 ± 1.59) (*p* = 0.046). Up to 30-year-old nondiabetics scored lower than more than 60-year-old nondiabetics (8.40 ± 1.5 versus 9.74 ± 2.03; *p* = 0.047). The youngest ate less olive oil (*p* = 0.002) and more pastries (*p* = 0.007).

**Conclusions:**

The sample presented moderate adherence to Mediterranean diet in all subgroups. Scientific evidence about the benefits of Mediterranean diet, olive oil, and nuts supports the recommendation to increase consumption of olive oil and nuts in diabetic women and of daily olive oil in type 2 diabetic men, reducing consumption of red meat, butter, and pastries, and to promote Mediterranean diet among the youngest of the sample studied.

## 1. Introduction

Lifestyle interventions, including diet control, have shown improvement in cardiovascular risk factors in patients with diabetes [1, 2]. Nutritional education in these patients has traditionally been based on low-fat diets and low-carbohydrate diets. Mediterranean diet, characterized by higher consumption of plant-based foods, comprises the following: high intake of fruits, vegetables, wholegrain products, legumes, fish, plant proteins, and unsaturated fatty acids (olive oil and nuts); moderate consumption of alcohol (mostly wine, preferably consumed with meals); and low consumption of (red) meat, commercial sweets or pastries, and low saturated fatty acids—fat [3].

Energy contributed by fat oscillates is between 25 and 35% of the total energy (calories), while energy provided by saturated fat is at most 7–8%. Important sources of fats are olive oil, nuts, seeds, and blue fish. Fat composition of Mediterranean diet has the following characteristics: high in monounsaturated fats, particularly oleic acid mainly from olive oil. Balanced in polyunsaturated fats (omega 6 and omega 3). Nuts and seeds are an important source of these fats. High consumption of long-chain omega-3 polyunsaturated fats (eicosapentaenoic acid and docosahexaenoic acid) from fish and olive oil (due to its content of linoleic and linolenic acids, precursors of arachidonic, eicosapentaenoic, and docosahexaenoic acids). Low in saturated fats, thanks to low consumption of butter and red meats. Absence of trans fats from industrial foods (margarines, fast food, and pastries).

Proteins account for about 15% of total calories. Proteins in Mediterranean diet come from various sources: legumes, nuts, white meats, fish and seafood, cheese, and yogurt. It is characterized by being relatively low in animal proteins.

Carbohydrates provide about 50% of total daily energy intake. Mediterranean diet is distinguished by being rich in complex carbohydrates, derived from cereals (bread, pasta), legumes, and various vegetable foods.

Glycemic index is considered quite low, due to large proportion of foods with low glycemic index, such as legumes, fruits, and vegetables, despite the consumption of other foods such as bread and honey (typical of Mediterranean diet) that have higher glycemic indexes.

Mediterranean diet is particularly rich in fiber and vegetable antioxidants, due to high consumption of various vegetable foods.

Relevant bibliography in recent years has demonstrated more favourable changes in fasting plasma glucose and insulin levels with moderately hypocaloric Mediterranean diet than with low-fat diet, and it has shown the same efficacy in weight loss in obese patients as low-carbohydrate or low-fat diets [4]. Furthermore, it has been proven that even a modest weight loss is related with an improvement in insulin sensitivity following a moderately hypocaloric Mediterranean diet [5].

In addition, Mediterranean diet reduces incidence of diabetes by 52% compared to a low-fat diet, in the absence of changes in body weight or physical activity [6, 7]. An Australian cohort study has shown reductions in total and diabetes mellitus-associated mortality in population (including European migrants) with greater adherence to a Mediterranean diet pattern [8].

Mediterranean diet enhanced with virgin olive oil and nuts was effective in inducing regression of carotid atherosclerosis among subjects with elevated baseline carotid intima-media thickness [9]. It has been shown that Mediterranean diet protects from the obesity risk allele action on the development of type 2 diabetes regardless of BMI [10]. Prevención con Dieta Mediterránea (PREDIMED) trial showed that Mediterranean diet supplemented with extra-virgin olive oil and nuts was useful to prevent cardiovascular disease in comparison with a low-fat diet [11]. Adherence to Mediterranean diet has been associated with lower incidence of cardiovascular disease regardless of the level of cardiovascular risk in a population with major cardiovascular risk factors [12]. Mediterranean dietary intervention may mitigate potential deleterious effects of elevated plasma ceramide concentrations (intermediate link between overnutrition and pathological mechanisms underlying cardiovascular disease) [13]. It is known that omega-3 polyunsaturated fatty acids, which are abundant in nuts, reduce triglyceride synthesis [14] and that olive oil has been associated with a greater amount of prostaglandin trienoic, which results in an increase in serum HDL-c [15].

Insulin resistance is the major determinant of type 2 diabetes mellitus. It is a multifactorial condition in which low-grade and chronic tissue inflammation is a major and documented contributor [16]. Type 1 diabetes is associated with increased risk of micro and macrovascular complications, and coronary artery disease is the main cause of death in type 1 diabetes. Type 1 diabetic population exhibits increased inflammation as evidenced by increased plasma C-reactive protein levels and increased monocyte proatherogenic activity [17].

Mediterranean diet has shown to play a role in reducing inflammatory state. Food antioxidants (fruits, vegetables, and nuts), reduction of saturated and trans fats, adequate omega-3 fatty acids, and dietary fiber intake have been suggested as Mediterranean diet components which may have anti-inflammatory roles [18].

Consumption of monounsaturated fatty acids in vivo has been related to improve insulin sensitivity [19]. Fish oils including omega-3 polyunsaturated fatty acids exert anti-inflammatory effects [20] through the inhibition of inflammasome (a component of the innate immune system) activation [21]. Inflammasome is a cytosolic protein complex assembled in response to pathogen infection or injury and promotes maturation and release of several proinflammatory cytokines.

Inflammatory response includes a process that produces a superfamily of chemical mediators that stimulate resolution of inflammatory responses. Specialized proresolving mediators are physiologic endogenous mediators and pharmacologic agonists that stimulate resolution of inflammation and infection. They include omega-3-derived families: resolvins, protectins, and maresins, as well as arachidonic acid-derived (n-6) lipoxins [22].

These biochemical pathways might explain at least in part the favourable effect of Mediterranean diet on glucose and insulin levels.

Metabolic syndrome, which can precede or partner with the development of type 2 diabetes mellitus, is also accompanied by a proinflammatory state associated with both insulin resistance and endothelial dysfunction. The link between inflammation and endothelial dysfunction, and subsequent cardiovascular disease, is oxidative stress. Inflammatory processes promote the production of reactive oxygen species which in turn stimulates mediators of proinflammatory state-like nuclear factor kappa B, cytokines, activator protein-1, vascular cell adhesion molecule 1, and intercellular adhesion molecule 1 [23].

Biochemical studies in the prevention mechanism of Mediterranean diet in atherosclerotic vascular disease have shown the main protection proceed from virgin olive oil and red wine. Hydroxytyrosol, oleuropein (olive oil polyphenols), resveratrol, and quercetin (red wine polyphenols) have shown to reduce inflammatory angiogenesis in vitro through reduced intracellular oxidative stress [24]. The complete polyphenol fraction from olive oil has been able to lower oxidative stress and inflammatory angiogenic responses associated with chronic degenerative diseases in endothelial cells [25].

Another complication highly prevalent in patients with diabetes mellitus is nonalcoholic fatty liver disease. Higher adherence to Mediterranean diet has been associated with less degree of insulin resistance and less severe liver disease among patients with nonalcoholic fatty liver disease [26], may be mediated by oleuropein, an olive oil's phenolic compound. Oleuropein-supplemented high fat diet-fed mice have shown less hepatic proinflammatory cytokines and less hepatic concentration of cholesterol, triglycerides, and free-fatty acids which have led to a more positive evolution of hepatic steatosis [27]. Thus, Mediterranean dietary pattern has been recommended for nonalcoholic fatty liver disease management [28].

These evidences attest to the interesting possibility that Mediterranean diet may play a significant role in the prevention of complications of both type 1 and type 2 diabetes mellitus. Therefore, to promote adherence to Mediterranean diet of diabetic population may be a key factor in the management of the disease.

### 1.1. Aims

The main purpose of this study was to identify adherence to Mediterranean diet among two groups of Spanish adults: diabetic patients and nondiabetic subjects.

Specifically, we aimed to analyse the differences between the two groups, due to educational efforts made to modify diabetic population eating habits and to identify the food groups to influence with nutritional education.

## 2. Materials and Methods

### 2.1. Design

A cross-sectional study was done with two participating groups selected from one Spanish medical centre population: one group of nondiabetic subjects and one group of diabetic patients.

### 2.2. Study Participants

Study participants (351) were a sample of volunteers recruited via nursing staff from the population of the Spanish medical centre “Grande Covian Specialty Medical Centre.”

Members of the population were asked if they would take part in the research when they attended usual medical centre activity and consultations and the information events held in the centre to mark the celebration of the May 28 World Nutrition Day.

Subjects who met the following criteria were eligible for the study: at least 18 years of age and given informed consent and male or female between the age of 18 and 90.

Subjects who met any of the following criteria were not eligible for the study: inability to fully comprehend and/or perform study procedures and subjects with very patent alteration of their cognitive functioning.

After advertising, 197 subjects volunteered for the diabetic group and 154 for the nondiabetic group.

The study was conducted in accordance with the Declaration of Helsinki (1964). The institution approved the study protocol, and all participants provided informed consent.

The sample size calculation indicated that the 351 subjects included in the study were sufficient to detect a difference of 0.6 points in the total score of the Mediterranean diet adherence screener between diabetic and nondiabetic subjects in a two-sided test, assuming a common standard deviation (SD) of 1.8 points with a significance level of 95% and a power of 80%.

### 2.3. Measurements

#### 2.3.1. 14-Item Mediterranean Diet Adherence Screener Score

Adherence to Mediterranean diet was measured by a 14-item Mediterranean diet adherence screener (Table 1), previously validated in adult Spanish population [29].

If the condition is not met, 0 points are recorded for the item. The final score ranges from 0 to 14; the greater the score, the greater the adherence to Mediterranean diet. Martínez-González et al. classified participants according to three Mediterranean diet adherence categories (≤5 points: low adherence, 6–9 points: moderate adherence, and ≥10 points: high adherence) [30].

### 2.4. Statistical Analysis

Analyses were performed with SPSS software package version 22.0 (SPSS Inc., Chicago, Illinois). Means (SD) or percentages were calculated for the 14-item dietary screener score across categories, and statistical significance of the differences was assessed with Mann–Whitney *U* test and chi-square test (if any of the cells had an expected count below 5, then Fisher exact test was applied). Comparisons were done between diabetic and nondiabetic subjects (separately for women and men), between type 1 diabetes and type 2 diabetes (separately for women and men), and between age groups (3 categories: 0–30/31–60 and 61–90 years) separately for diabetic and nondiabetic subjects.

Spearman rank correlations were calculated between the duration of diabetes and the 14-item score, for the whole diabetic group and separately for type 1 diabetic subjects and type 2 diabetic subjects.

Nonparametric tests were chosen because the dependent variable showed a nonnormal distribution. Statistical significance was set at two-sided *p* < 0.05. We did not use any imputation method for missing data.

## 3. Results

Mean (±SD) age of the whole sample was 56.25 ± 13.6 years, in type 1 diabetic subjects was 46.78 ± 12.13, in nondiabetic subjects was 50.97 ± 12.58, and in type 2 diabetic subjects was 64.42 ± 10.87. Age differences among study subgroups are shown in Table 2.

For the whole sample, 14-item dietary screener score mean (±SD) was 8.77 ± 1.82 points on the 0 to 14 score. The 14-item score in nondiabetic subjects was 9.06 ± 1.78 and in diabetics was 8.54 ± 1.81.

We did not observe significant correlations in the 14-item score depending on the duration of diabetes (Table 3).

Nondiabetic participants scored higher on the total 14-item score than diabetics (*p* = 0.006). There were also differences between men and women with diabetes (*p* = 0.023) (Table 4). There were no differences between nondiabetic men and women. Percentages in adherence categories to Mediterranean diet for each participant subgroup can be observed in Table 4.

There were no differences between type 1 and 2 diabetics (Table 5).

Diabetic women (8.15 ± 1.79) showed differences with nondiabetic women (9.19 ± 1.84) (*p* = 0.003), both type 1 (7.92 ± 1.6) (*p* = 0.012) and type 2 (8.12 ± 1.94) (*p* = 0.005).

Among men subgroups, there were no differences (*p* = 0.08).

**Figure 1:**
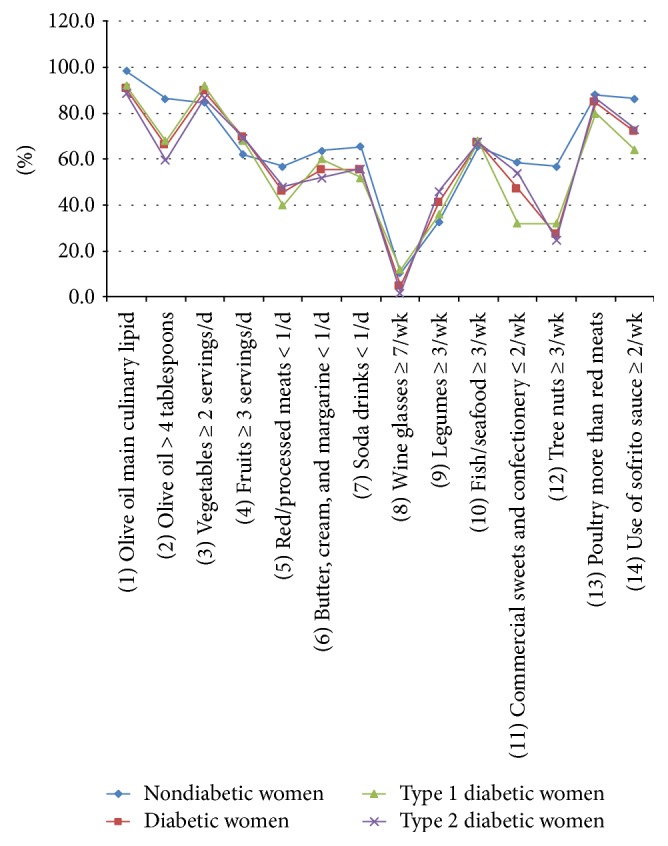
Percentage of women with positive criteria for 1 point in each of the 14 items of the dietary screener, in all women subgroups.

Figure 1 reflects the percentage of women with positive criteria for 1 point in each of the 14 items of the dietary screener, in all women subgroups.

**Figure 2:**
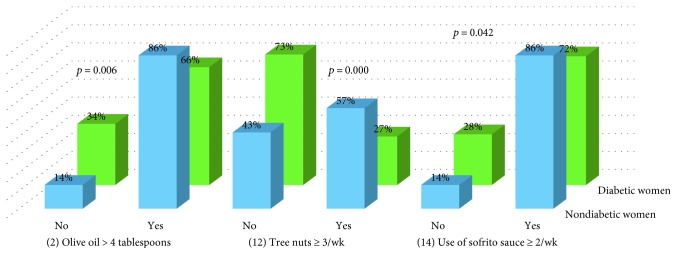
Comparison of items between nondiabetic women and diabetic women.

Comparison showed differences in items 2 (*p* = 0.006), 12 (*p* = 0.000), and 14 (*p* = 0.042) between nondiabetic women and diabetic women (Figure 2).

**Figure 3:**
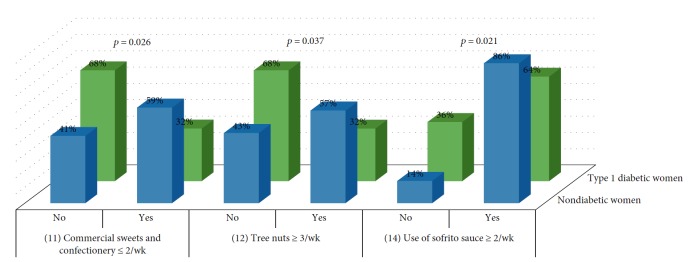
Comparison of items between nondiabetic women and type 1 diabetic women.

**Figure 4:**
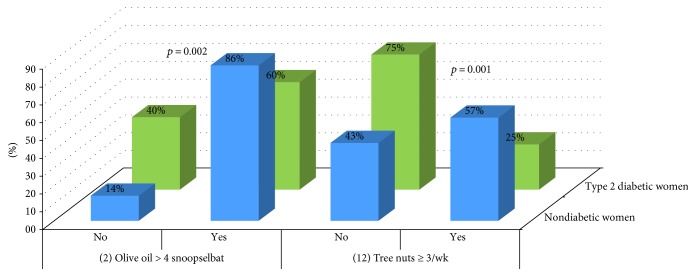
Comparison of items between nondiabetic women and type 2 diabetic women.

Analysis showed differences between type 1 diabetic women and nondiabetics in items 11 (*p* = 0.026), 12 (*p* = 0.037), and 14 (*p* = 0.021) (Figure 3) and between type 2 diabetic women and nondiabetics in items 2 (*p* = 0.002) and 12 (*p* = 0.001) (Figure 4).

**Figure 5:**
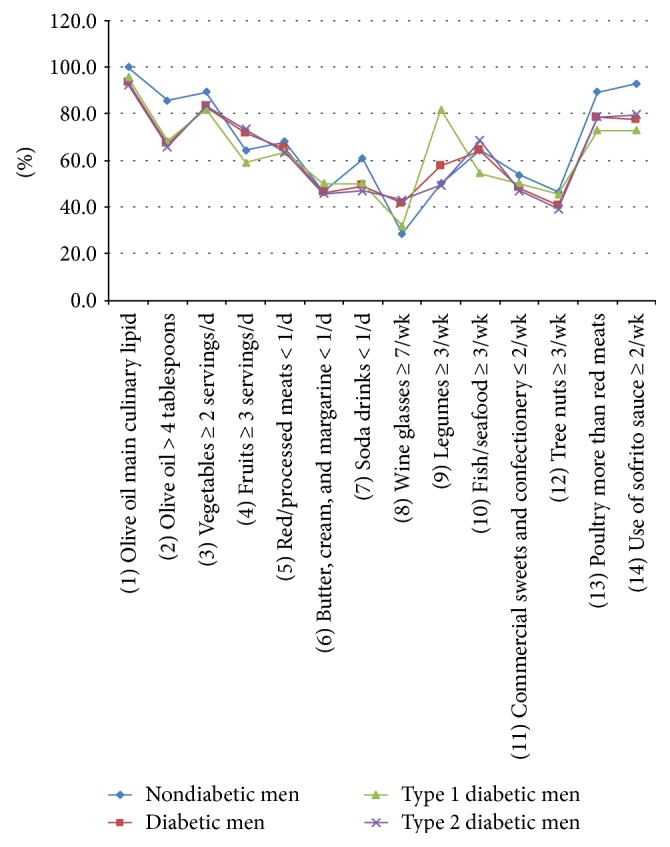
Percentage of men with positive criteria for 1 point in each of the 14 items of the dietary screener, in all men subgroups.

Figure 5 reflects the percentage of men with positive criteria for 1 point in each of the 14 items of the dietary screener, in all men subgroups.

Comparison did not show differences between nondiabetic men and diabetic men.

**Figure 6:**
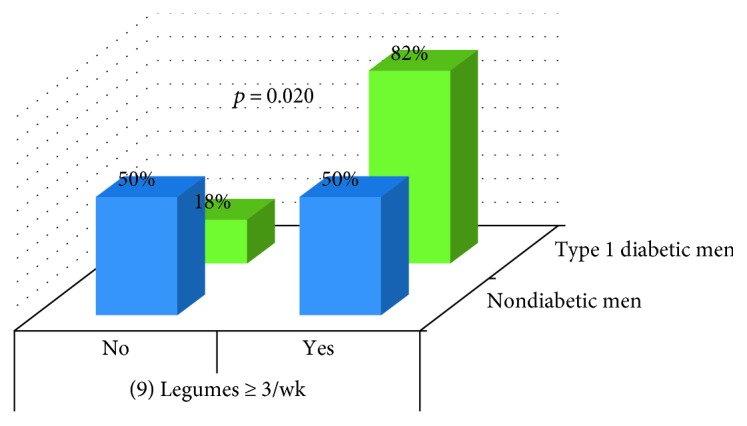
Comparison of items between nondiabetic men and type 1 diabetic men.

**Figure 7:**
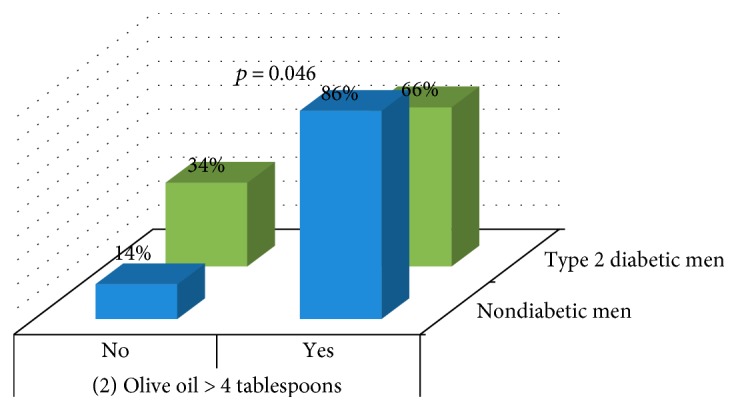
Comparison of items between nondiabetic men and type 2 diabetic men.

Analysis showed differences between type 1 diabetic men and nondiabetics in the item 9 (*p* = 0.020) (Figure 6) and between type 2 diabetic males and nondiabetics in the item 2 (*p* = 0.046) (Figure 7).

**Figure 8:**
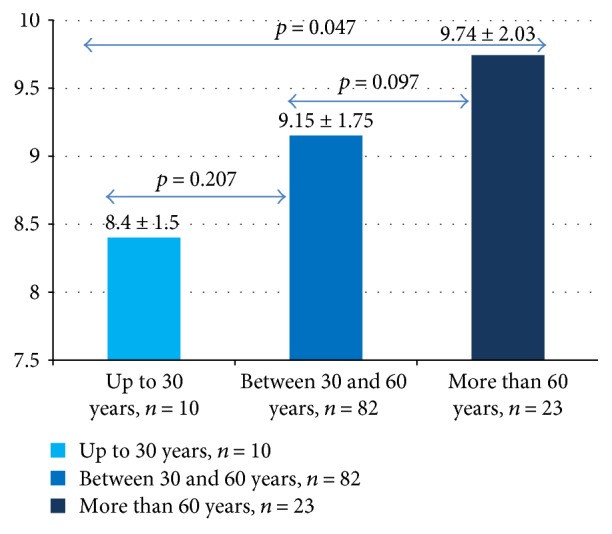
Differences between the 14-item score in age groups of nondiabetic subjects.

When we compared the 14-item dietary screener score between age groups in nondiabetic subjects (3 categories: 0–30/31–60 and 61–90 years), the youngest group scored lower than the oldest group (*p* = 0.047) (Figure 8).

**Figure 9:**
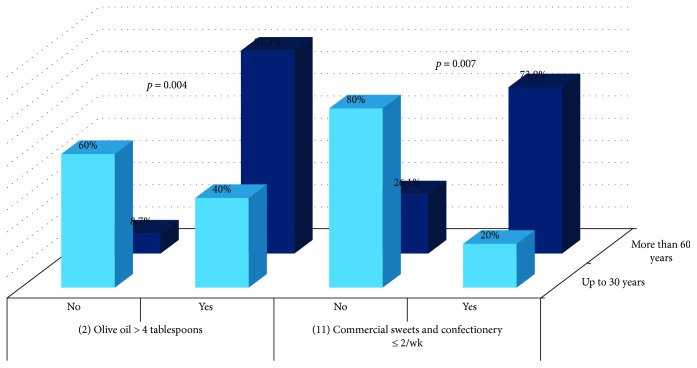
Comparison of items between the youngest and the oldest nondiabetic participants.

Analysis showed differences between the youngest and the oldest in the item 2 (*p* = 0.004) and in the item 11 (*p* = 0.007) (Figure 9).

When we compared the 14-item dietary screener score between age groups in diabetic subjects, no differences were observed.

## 4. Discussion

For the whole sample, the 14-item dietary screener score (8.77) was similar to the score recorded by Ortega-Azorín et al. (8.70) [10] in Spanish high-cardiovascular risk subjects with type 2 diabetes and nondiabetics, but better than that observed by Munoz-Pareja et al. (6.83) [31] in the same year in a representative sample of noninstitutionalized Spanish population aged 18 years and over.

Adherence to Mediterranean diet in nondiabetic subjects was 9.06 and in diabetics was 8.54 (type 1 diabetic patients: 8.30; type 2 diabetic patients: 8.48). Nondiabetics had much greater adherence to Mediterranean diet than the sample studied by Salas-Salvado et al. of nondiabetic individuals at high cardiovascular risk (8.2) [6] and the nondiabetic subjects studied by Ortega-Azorín et al. (8.7) [10]. Our type 2 diabetic patients (8.48) exhibited similar adherence to that found by Ortega-Azorín et al. in type 2 diabetic patients (8.5) [10]. Our type 1 diabetic patients (8.30) showed less adherence than that found by Carral et al. (8.9) [32].

Mean indicated moderate adherence to Mediterranean diet in all subgroups. The majority of patients in all subgroups had moderate adherence to Mediterranean diet, except in the nondiabetic men subgroup, in which, 57.1% had high adherence.

We did not observe significant correlations in the 14-item score depending on the duration of diabetes. Similar results were found by Ortega-Azorín et al. [10].

Diabetics showed less adherence to Mediterranean diet than the nondiabetic subjects. Diabetic women showed lower adherence than diabetic men. In fact, women generated lower adherence among diabetics since there were no differences between diabetic and nondiabetic men in the total score.

Traditional nutritional education for diabetics based on low-fat diet [4, 33], which promotes weight loss [4, 34], may be related to the lower adherence shown by type 2 diabetic women to Mediterranean diet in items “2: olive oil > 4 tablespoons” and “12: tree nuts ≥ 3/week” and by type 2 diabetic men in the item “2: olive oil > 4 tablespoons.” Similar reasons may be related to the lower adherence shown by type 1 diabetic women to Mediterranean diet on issues “12: tree nuts ≥ 3/week” and “14: use of sofrito sauce ≥ 2/week,” although despite nutritional education for diabetics, they ate more pastries than nondiabetic women. However, some good results had been obtained by nutritional education. Better punctuations have been obtained by type 1 diabetic men in the answer to question “9: How many servings of legumes do you consume per week? (1 serving: 150 g).”

These worst results obtained by type 2 diabetics clearly showed efforts made to reduce body weight, following a low-fat diet, but diminishing the adherence to Mediterranean diet by reducing the intake of olive oil and nuts without reducing the intake of red meat or butter (similar percentages between diabetic and nondiabetic individuals).

Our results suggest that future nutritional education programs should recommend an increase in the consumption of nuts and olive oil in the diabetic women and of olive oil in the type 2 diabetic men. Recommendations should take into account the total fat intake of the diet in order to keep to the 35% calories from fat, which implies reducing fat intake from other sources such as red meat, butter, or pastries.

Being overweight, having abdominal fat distribution, and being obese are the most important risk factors for type 2 diabetes mellitus [35], and increased fat mass contributes to inflammation [36]. The need to maintain a healthy weight is a nonsignificant factor.

Itsiopoulos et al. [37] provided a 12-week normocaloric Mediterranean diet (energy mean 2.600 kcal per day), high in fat (40% of energy; >50% from monounsaturated fats), moderate carbohydrate (44% of energy), moderate protein (12% of energy), moderate alcohol (4% of energy from red wine) with a large quantity of fruits (563 g/day) and vegetables (691 g/day), and olive oil (75 ml/day) to type 2 diabetic adults. After 12 weeks, a trend towards weight loss was observed (body mass index: −0.3). Higher postprandial fat oxidation rate and higher thermic effect of food following a meal high in monounsaturated fatty acid [38] and higher dietary fibre intake due to large quantities of fruits, vegetables, and legumes [39] may partly explain the trend towards weight loss.

To these benefits of a normocaloric Mediterranean diet, those of a moderately hypocaloric Mediterranean diet may be added. A hypocaloric Mediterranean diet being equal to the resting metabolic rate (mean 1.500 kcal per day) has favoured weight loss (mean 4.9 ± 0.9 kilograms) in obese women after 2 months [40], and a 12-week hypocaloric Mediterranean diet based on the restriction of 40% of habitual energy intake has led to 6-kilogram weight loss, in patients with metabolic syndrome [41]. Shai et al. in 2008 prescribed a moderate-fat, restricted-calorie (1500 kcal per day for women and 1800 kcal per day for men) Mediterranean diet to moderately obese subjects. The maximum weight reduction (−4.4 ± 6.0 kg) was achieved during the first 6 months; this period was followed by the 24-month maintenance phase. Weight loss 24 months postintervention was more sustainable on Mediterranean diet than on low-fat or low-carbohydrate diet [4]. Recently, a personalized Mediterranean hypocaloric diet (1400–1600 kcal per day) brought to an average reduction of body weight of 5.8% at 4 months, with an improvement in insulin sensitivity [5].

Spain is included in low cardiovascular risk countries in Europe [42]. Life expectancy in Spain is 10 years older than the world average, and in both sexes, Spain remains within the top five positions worldwide [43]. Mediterranean diet might be one of the factors by which Spain maintains low levels of cardiovascular risk [2]. The youngest among our nondiabetic subjects had less adherence to Mediterranean diet than the oldest ones. They consumed less olive oil and more pastries. There is a growing evidence that the elderly people living in Mediterranean area seem to better hold the traditional healthy dietary habits than the younger ones [3, 44, 45]. These results point to the decrease in adherence to Mediterranean diet of the youngest, which may contribute to increase their lifetime risk of chronic disease.

Today, economic, social, demographic, and health factors are associated with a more “Westernized” type of diet [46]. Industrialized countries, in the last decades, have lost much of their native agricultural production which makes difficult the arrival in the market of fresh and healthy products. The 2030 Agenda for Sustainable Development, adopted by world leaders in September 2015, at a United Nations summit, has identified 17 sustainable development goals, four of which can be addressed by sustainable food consumption and sustainable diets. Sustainable diet was defined in 2010 including two perspectives: a nutrition perspective, focused on individuals, and a global sustainability perspective, in all its dimensions: environmental, economic, and social [47]. Sustainable diets should promote health and well-being of populations, while being protective of the environment and preserving of its resources [48]. Local agriculture in industrialized countries should be supported. Not all countries grow the same fruits and vegetables, but with the current technological advances and respecting the species and biodiversity of each climate, in all countries, there could be a greater, better, and cheaper supply of fruits and vegetables. Countries should be encouraged to provide their populations with protective foods that satisfy both components of health and sustainability.

In Spain, the pattern of traditional Mediterranean diet is being lost. Health professionals must encourage the education and promotion of healthy habits linked to traditional gastronomy in Mediterranean area. Adoption of a diet by individuals is a part of the change in the food system, and adoption of sustainable diets can be facilitated by proper education.

Available scientific evidence on benefits of Mediterranean diet supports transmission of these healthy habits from health professionals to the youngest and to patients that could benefit greatly, such as the diabetic population.

## 5. Limitations

This research has limitations that should be considered. Diagnosis of diabetes was self-reported, which means that reporting bias cannot be excluded. Our sample may not be representative of the general Spanish population. It was composed of adults and elderly from a single urban location, so it may not be a representative of people from other geographical areas. However, our findings may be relevant for the development of recommendations for population-based approaches to Mediterranean diet.

## 6. Conclusions

Diabetic women had lower adherence to Mediterranean diet than nondiabetic women. Diabetic women consumed less olive oil and less nuts than the nondiabetic ones. Type 2 diabetic men consumed less olive oil than nondiabetic men. The youngest nondiabetic individuals had less adherence to Mediterranean diet than the older ones. The sample studied presented moderate adherence to Mediterranean diet in all subgroups. Scientific evidence about the benefits of Mediterranean diet, olive oil, and nuts supports the recommendation to increase consumption of olive oil and nuts in diabetic women and of daily olive oil in type 2 diabetic men, reducing consumption of red meat, butter, and pastries, and to promote Mediterranean diet among the youngest of the sample studied.

## Figures and Tables

**Table 1:** 14-item Mediterranean diet adherence screener.

Items	Criteria for 1 point
(1) Do you use olive oil as main culinary fat?	Yes
(2) How much olive oil do you consume in a given day (including oil used for frying, salads, and out-of-house meals)?	≥4 tablespoons (1 tablespoon = 13.5 g)
(3) How many vegetable servings do you consume per day? (1 serving: 200 g (consider side dishes as half a serving))	≥2 (≥1 portion raw or as a salad)
(4) How many fruit units (including natural fruit juices) do you consume per day?	≥3
(5) How many servings of red meat, hamburger, or meat products (ham, sausage, etc.) do you consume per day? (1 serving: 100–150 g)	<1
(6) How many servings of butter, margarine, or cream do you consume per day? (1 serving: 12 g)	<1
(7) How many sweet or carbonated beverages do you drink per day?	<1
(8) How much wine do you drink per week?	≥7 glasses
(9) How many servings of legumes do you consume per week? (1 serving: 150 g)	≥3
(10) How many servings of fish or shellfish do you consume per week? (1 serving 100–150 g of fish or 4-5 units or 200 g of shellfish)	≥3
(11) How many times per week do you consume commercial sweets or pastries (not homemade), such as cakes, cookies, biscuits, or custard?	<3
(12) How many servings of nuts (including peanuts) do you consume per week? (1 serving 30 g)	≥3
(13) Do you preferentially consume chicken, turkey, or rabbit meat instead of veal, pork, hamburger, or sausage?	Yes
(14) How many times per week do you consume vegetables, pasta, rice, or other dishes seasoned with sofrito (sauce made with tomato and onion, leek, or garlic and simmered with olive oil)?	≥2

**Table 2:** Age in study subgroups.

	Age	Mean	SD	*p*
Women	Type 1 diabetic subjects	44.13	12.00	0.003
	Nondiabetic subjects	50.34	10.05	
				0.000
	Type 2 diabetic subjects	66.33	12.20	

Men	Type 1 diabetic subjects	49.68	11.72	0.014
	Nondiabetic subjects	57.11	11.52	
				0.021
	Type 2 diabetic subjects	63.33	9.97	

**Table 3:** Spearman rank correlations between the 14-item score and the duration of diabetes.

	Duration of diabetes	14-item screener score	*n*	Spearman's rho
Diabetic patients	15.67	8.54	165	0.008
Type 1 diabetic patients	19.31	8.3	45	−0.038
Type 2 diabetic patients	14.3	8.48	119	0.033

**Table 4:** Comparison of 14-item dietary screener score between nondiabetic subjects and diabetic patients.

14-item screener score	*n*	Mean	SD	Categories of adherence to Mediterranean diet %		*p*	Gender	*n*	Mean	SD	Categories of adherence to Mediterranean diet %		*p*
Nondiabetics	154	9.06	1.78	Low	2.6	0.006	Women	58	9.19	1.84	Low	1.70	0.568
											Moderate	55.20	
											High	43.10	
				Moderate	52.60		Men	28	9.36	1.59	Low	0.00	
				High	44.80						Moderate	42.90	
											High	57.10	
Diabetic patients	197	8.54	1.81	Low	4.10		Women	85	8.15	1.79	Low	7.10	0.023
											Moderate	70.60	
				Moderate	69.00						High	22.40	
				High	26.90		Men	106	8.82	1.75	Low	1.90	
											Moderate	67.90	
											High	30.2	

**Table 5:** Comparison of 14-item dietary screener score between type 1 diabetic patients and type 2 diabetic patients.

14-item screener score	*n*	Mean	SD	*p*	Gender	*n*	Mean	SD	*p*
Type 1 diabetic patients	47	8.30	1.46	0.967	Women	25	7.92	1.60	0.061
					Men	22	8.73	1.16	
Type 2 diabetic patients	132	8.48	1.92		Women	52	8.12	1.94	0.082
					Men	79	8.76	1.88	
